# Evidence for early and late prediction error-associated ERP responses during probabilistic cueing

**DOI:** 10.1016/j.isci.2026.117545

**Published:** 2026-09-18

**Authors:** Insa Schlossmacher, Alena Schlotmann, Sebastian Schindler, Robert Moeck, Thomas Straube

**Affiliations:** 1 Institute of Medical Psychology and Systems Neuroscience, University of Münster, 48149 Münster, Germany; 2 Otto Creutzfeldt Center for Cognitive and Behavioral Neuroscience, University of Münster, 48149 Münster, Germany

**Keywords:** probabilistic cueing, expectation suppression, surprise enhancement, attention, ERPs

## Abstract

Probabilistic relationships are omnipresent in our sensory environment. According to some predictive coding models, the processing of deviations from expectation should be accompanied by enhanced neuronal responses, reflecting a prediction error, while expected stimuli should lead to suppression of brain responses. However, probabilistic cueing designs show no clear picture regarding prediction error and expectation suppression. Here, we measured event-related potentials (ERPs) while participants were presented with a probabilistic cueing procedure with low, high, or equal-probable stimuli, while the task relevance of the probabilistic relations was varied. Our data show enhanced ERPs to infrequent stimuli in early (increased negative amplitudes over posterior electrodes) and late (increased positive amplitudes over central electrodes) time windows, driven by a prediction error and not by expectation suppression. While early visual responses are independent of task-relevance of predictive information, later and hierarchically higher effects are potentiated if predictive information is explicitly attended.

## Highlights

• We measured EEG in a visual cueing design varying attention to predictive relations
• We found early as well as late increased responses to unexpected stimuli
• Effects were driven by surprise enhancement and not expectation suppression
• Attention potentiated late effects but not early ones

## Graphical abstract

**Figure:**
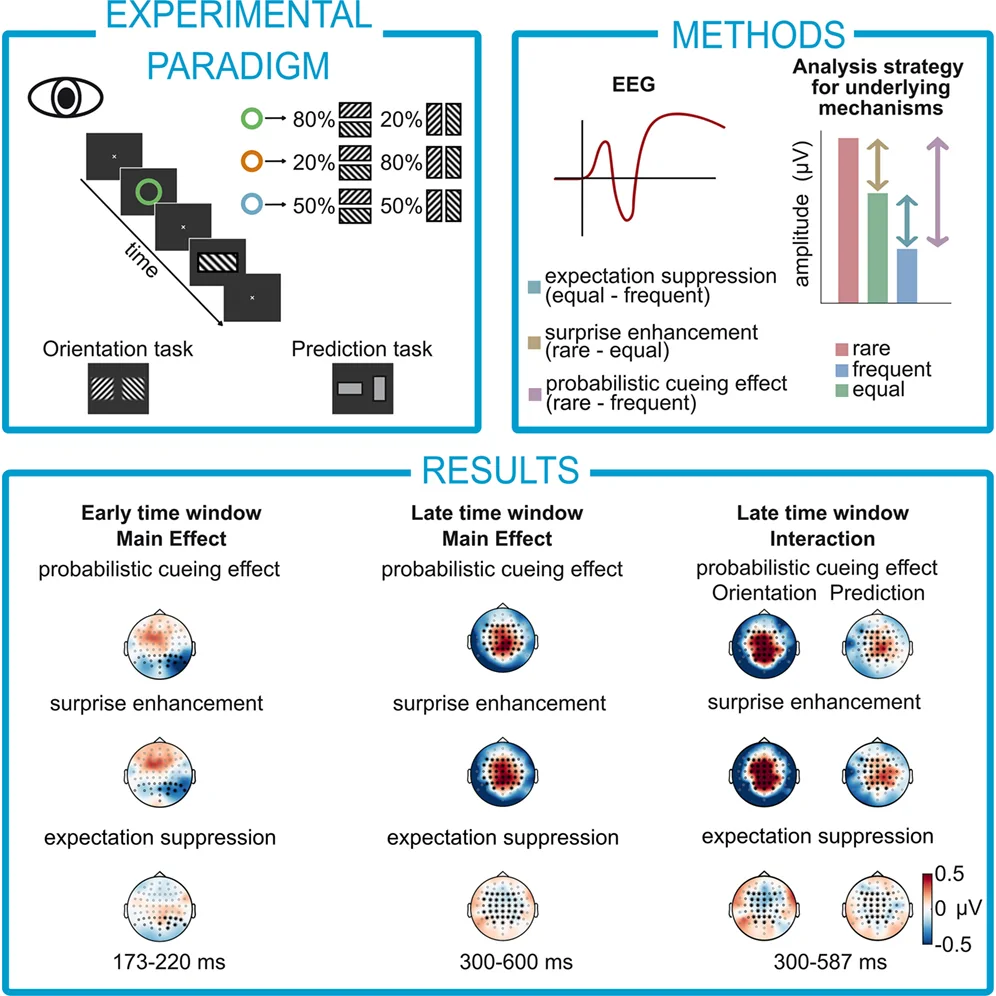

## Teaser

Cognitive neuroscience; Techniques in neuroscience

## Introduction

Learning probabilistic relations between consecutive events in our environment is important. For example, one might expect the traffic light to change color in a predictable fashion, e.g., from green to orange to red. The impact of clear predictions (i.e., orange is followed by red) can be investigated experimentally, using cue-based learning where two stimuli are paired with different co-occurrence probabilities. Predictive processing accounts offer a theoretical underpinning for differences in responsiveness to more or less probable stimuli. From this point of view, the brain generates a model of the world against which the incoming sensory data are compared. Higher levels supposedly generate predictions that are passed down and lower levels compute a prediction error (PE) that is passed upwards.^1^^,^^2^^,^^3^^,^^4^^,^^5^

However, while theoretic accounts suggest that predictive coding leads to both suppressed and enhanced activity depending on predictive information,^1^^,^^2^^,^^3^^,^^4^^,^^5^ the simple difference between expected and unexpected events could be due to both phenomena, i.e., to reduced response to expected stimuli (= expectation suppression) or to increased activity to unexpected ones (= surprise enhancement).^6^ While fMRI studies show smaller responses for expected compared to unexpected stimuli in cue-based learning paradigm,^7^^,^^8^^,^^9^^,^^10^^,^^11^^,^^12^ it remains unclear whether these effects are caused by expectation suppression or surprise enhancement. The inclusion of an equal-probability control condition^6^^,^^7^^,^^9^ or representational similarity analyses with deep neural networks representing PEs^11^^,^^12^ are suggested to allow the investigation of the underlying mechanisms. While most studies using these approaches found evidence for surprise enhancement, i.e., PEs, the evidence for expectation suppression is less clear.^7^^,^^8^^,^^9^^,^^11^^,^^12^

Surprisingly, there is no clear picture regarding probabilistic cueing effects in human electroencephalography (EEG) and magnetoencephalography (MEG) studies. Two studies observed significant expected-unexpected differences,^13^^,^^14^ which were likely driven by surprise enhancement.^13^ In contrast, several studies could not replicate this finding. Den Ouden et al.^15^^,^^16^ and Hu et al.^17^ observed no event-related potential (ERP) differences between expected and unexpected stimuli, leading them to conclude that visual probabilistic cueing effects are not automatically evoked. In addition, Zhou et al.^18^ and Kok et al.^19^ did not observe effects on event-related fields if cue-expectations were violated. Nevertheless, evidence of differences in neural responses to expected and unexpected stimuli were provided via decoding^16^^,^^17^^,^^19^^,^^20^ and time frequency analyses.^18^

Remarkably, this pattern of results conflicts with the repeatedly demonstrated evidence of visual ERP mismatch effects in oddball paradigms.^21^^,^^22^^,^^23^ While in the oddball paradigm, effects are often confounded with stimulus repetition and adaptation to high-frequent stimuli,^6^ there is evidence for a genuine mismatch negativity (MMN) driven by surprise enhancement, i.e., a PE, during early time windows across sensory modalities.^23^ Furthermore, several studies suggested that PE responses, in contrast to simple adaptation effects, depend on attention or awareness,^24^^,^^25^ which points to the role of task conditions and processing resources to observe effects of prediction on infrequent stimuli.^26^

Thus, the evidence for early PE in MMN studies contrasts with the reported absence of effects in probabilistic cueing studies. Reasons for this discrepancy could be found in the complexity of cueing designs, the insufficient attention to the cue or the fundamental difference between the two approaches to investigate mismatch responses. In the current study, we investigated the effects of probabilistic cueing on ERP responses to cued stimuli with a suited design. Participants were presented with different-colored circles as cues, followed by horizontal and vertical rectangles that varied in their probabilistic relationships with the cues (Figure 1A). To characterize predictive cueing effects and distinguish surprise enhancement from expectation suppression, we included two cues with an 80:20 mapping and one with a 50:50 equiprobable mapping resulting in rare, frequent and equal probable stimuli (Figure 1B). On the one hand, surprise enhancement was quantified by the difference between rare and equal stimuli as the rare stimulus was unexpected compared to the equal probable one. While on the other hand, expectation suppression was quantified by the difference between equal and frequent stimuli as the frequent stimuli was expected compared the equal probable one, see Figure 1C. To test the effects of attention, participants attended to cue-target information in one block (prediction task) and to an association-unrelated feature (orientation task) in another (Figure 1A).

**Figure 1:**
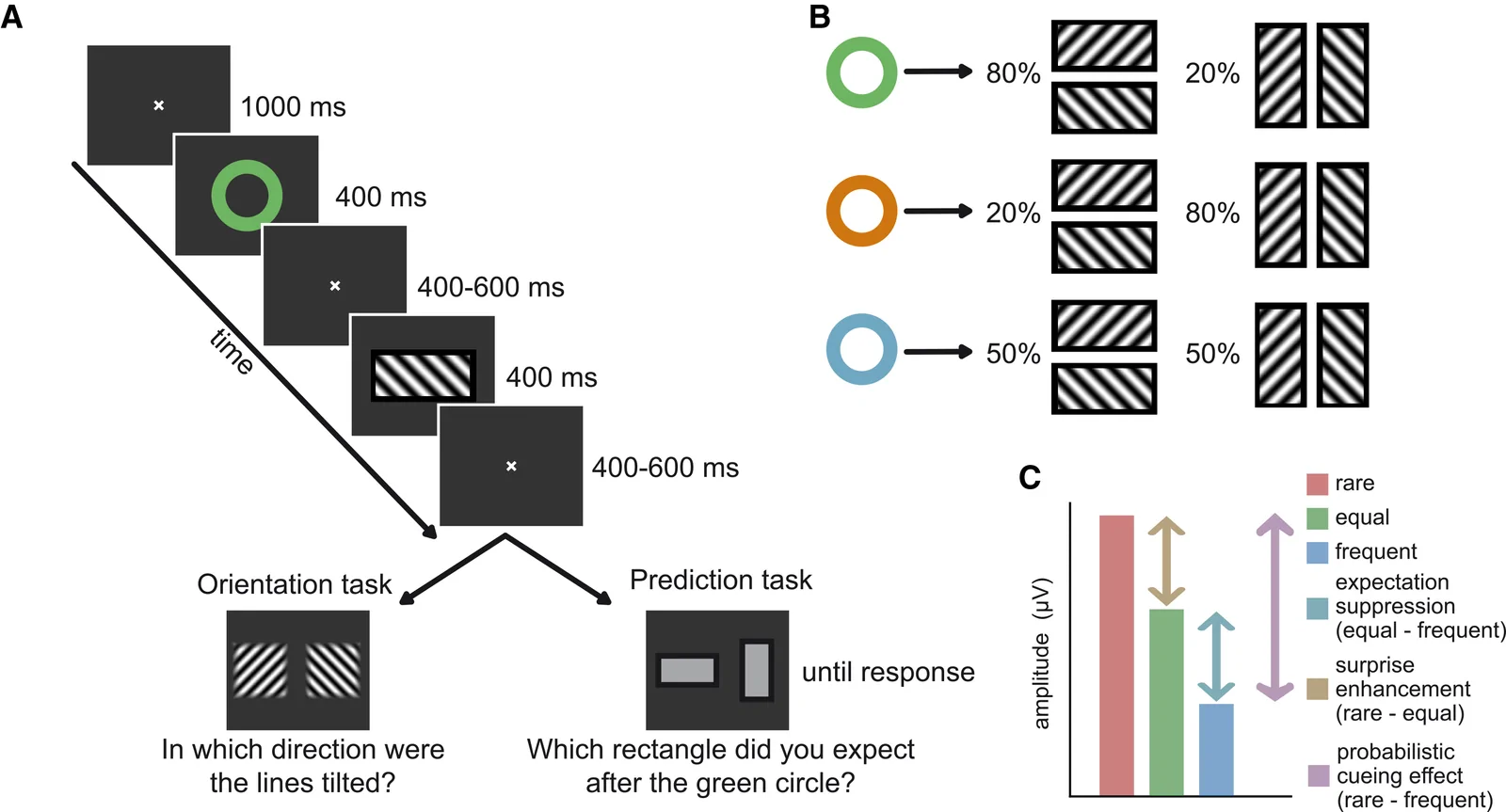
Experimental setup (A) Trial sequence with sequential presentation of colored circle and rectangle. In 20% of the trials a question followed the presentation of the rectangle. In one block participants had to attend the line orientation, in another block the shape. (B) One possible mapping for colored circles to rectangle shapes. (C) Decomposition of observed probabilistic cueing effects into expectation suppression and surprise enhancement.

## Results

One participant had to be excluded due to a high number of rejected trials in their EEG data (≥40%). With regard to the remaining 39 participants, on average, 17.32% (SD = 7.02%) of trials were excluded from the analysis due to artifacts and on average 0.77 (SD = 0.93) electrodes were interpolated. A repeated measures ANOVA indicated no difference in percentage of excluded trials for the factor probability (*F*[2, 76] = 0.04, $\hat{\varepsilon }$ = 0.81, *p* = 0.94), task (*F*[1, 38] = 0.54, *p* = 0.47) and the interaction (*F*[2, 76] = 1.23, *p* = 0.30). We averaged all available trials for each condition (prediction task: N_rare_ = 52.97 [6.09], N_equal_ = 131.85 [13.18], N_freq_ = 209.85 [19.15]; orientation task: N_rare_ = 53.31 [6.19], N_equal_ = 133.67 [14.51], N_freq_ = 214.56 [24.20]). Sphericity was violated in the early as well as the late time window when comparing cluster averages in the different probability conditions (early main effect: *w* = 0.73, *p* = 0.003, late main effect: *w* = 0.55, *p* < 0.001, late interaction effect: *w* = 0.60, *p* < 0.001). Applying the sphericity correction by Greenhouse-Geisser did not change the significance of the effects.

### Impact of probability conditions on task performance depends on task relevance

Participants were able to easily perform the prediction task, as evidenced by the percentage of matches between the presented rectangle and button press in (Figure 2A). For example, if a horizontal rectangle was presented in the frequent condition (i.e., it was predicted by the cue), participants pressed the horizontal button. In contrast, when the horizontal rectangle was presented in the rare condition (i.e., not predicted by the cue), participants pressed the vertical button. Thus, the high number of matches in the frequent condition, the about 50% matches in the equal condition, and the low number of matches in the rare condition correspond to the cue-induced expectations. Repeated measures ANOVA in the prediction task indicated a significant effect of condition (*F*[2, 76] = 1530.45, $\hat{\varepsilon }$ = 0.72, *p* < 0.001), see Figure 2A. Post hoc *t*-tests indicated a significant difference in the percentage of matches between all conditions (all *p*_*holm*_*s* < 0.001, all BFs >3). Repeated measures ANOVA on median reaction times in the prediction task indicated a significant effect of condition (*F*[2, 76] = 10.97, *p* < 0.001), see Figure 2A. Post-hoc *t*-tests indicated higher reaction time in the rare compared to the frequent condition (*t*[38] = −2.38, *p*_*holm*_ = 0.04, BF_10_ = 2.09), as well as a higher reaction time in the equal compared to rare (*t*[38] = −2.17, *p*_*holm*_ = 0.04, BF_10_ = 1.40) and frequent (*t*[38] = −5.08, *p*_*holm*_ < 0.001, BF_10_ = 1,958.40) condition.

**Figure 2:**
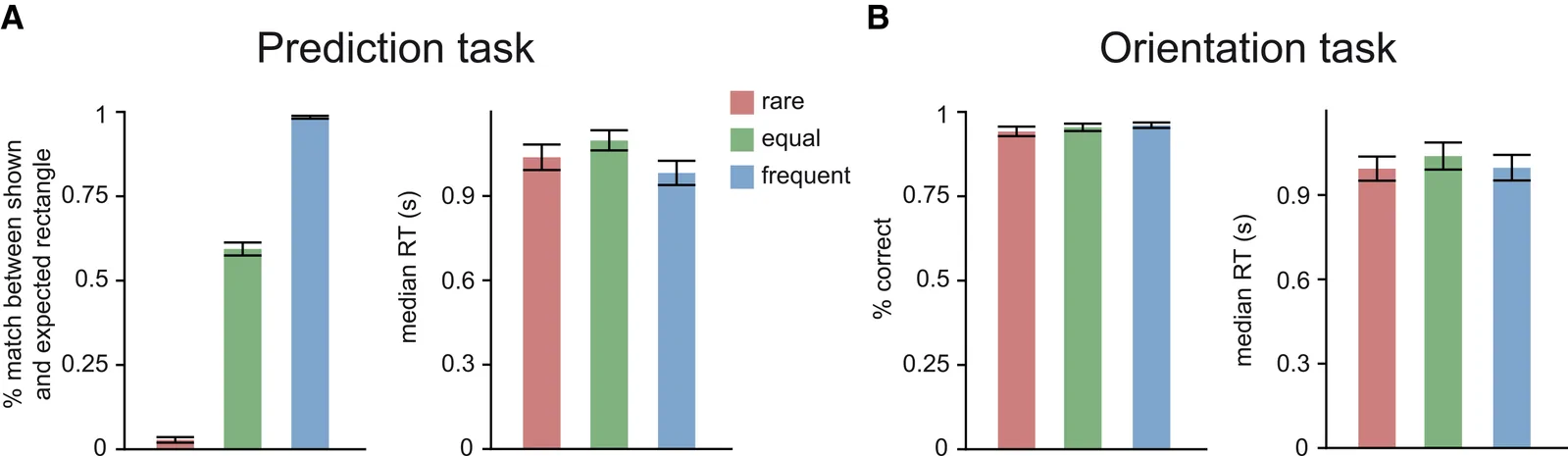
Behavioral data (A) Percentage of trials where button presses matched with the presented rectangle and median reaction times in the rare, frequent and equal condition in the prediction task. Note that for the frequent condition participants correctly presses the button corresponding to the presented rectangle, while in the rare condition they correctly pressed the button that did not match the presented rectangle. (B) Hit rates and median reaction times in the orientation task. Error bars correspond to standard error of the mean.

In the orientation task, there was no significant effect of condition (*F*[2, 76] = 1.73, *p* = 0.18) on hit rates, see Figure 2B. Repeated measures ANOVA on median reaction times in the orientation task indicated a significant effect of condition (*F*[2, 76] = 4.81, $\hat{\varepsilon }$ = 0.83, *p* = 0.02), see Figure 2D. Post-hoc *t*-tests indicated slower reaction times in the equal condition compared to the rare and frequent one (equal vs. rare: *t*[38] = 2.88, *p*_*holm*_ = 0.01, BF_10_ = 5.98; equal vs. frequent: *t*[38] = 3.21, *p*_*holm*_ = 0.008, BF_10_ = 12.64), while evidence for the absence was found comparing rare and frequent condition (*t*[38] = 0.18, *p* = 0.86, BF_01_ = 5.701).

### Early negativities indicate surprise enhancement independent of task relevance

Cluster-based permutations revealed a significant main effect of probability (sum[*F*] = 522.50, *p* = 0.04) extending from 173 to 220 ms with a temporal peak at 196 ms. Post-hoc *t*-tests indicated that cluster averages of rare stimuli were significantly more negative compared to both frequent and equal condition (rare vs. frequent: *t*[38] = −3.93, *p*_*holm*_ = 0.001, BF_10_ = 78.72; rare vs. equal: *t*[38] = −3.49, *p*_*holm*_ = 0.004, BF_10_ = 25.11), while evidence for the absence of an effect between equal and frequent condition was obtained (*t*[38] = −0.24, *p*_*holm*_ = 0.81, BF_01_ = 5.63). See the left column in Figures 3A–3C for a visualization of the results. Importantly, this pattern could be observed in the orientation (rare vs. frequent: *t*[38] = −2.96, *p*_*holm*_ = 0.02, BF_10_ = 7.14; rare vs. equal: *t*[38] = −2.48, *p*_*holm*_ = 0.04, BF_10_ = 2.54; equal vs. frequent: *t*[38] = −0.30, *p*_*holm*_ = 0.77, BF_01_ = 5.55) as well as the prediction task (rare vs. frequent: *t*[38] = −2.85, *p*_*holm*_ = 0.01, BF_10_ = 5.51; rare vs. equal: *t*[38] = −3.04, *p*_*holm*_ = 0.01, BF_10_ = 8.54; equal vs. frequent: *t*[38] = −0.02, *p*_*holm*_ = 0.98, BF_01_ = 5.79), see Figure 4. Furthermore, we found two clusters for a significant main effect of task. The first cluster (sum[*F*] = 1,848.77, *p* = 0.02) extended from 100 to 165 ms with a temporal peak at 134 ms. The second cluster (sum[*F*] = 19,658.57, *p* < 0.001) extended from 157 to 300 ms with a temporal peak at 243 ms. Post-hoc *t* tests indicated that cluster averages of the first cluster were more positive in the prediction task than the orientation task (*t*[38] = −5.26, *p* < 0.001, BF_10_ = 3,329.97), while the reverse was true for the second cluster (*t*[38] = 8.01, *p* < 0.001, BF_10_ = 10.24×10^6^). No significant interaction was found (all clusters’ uncorrected *p*s > 0.25).

**Figure 3:**
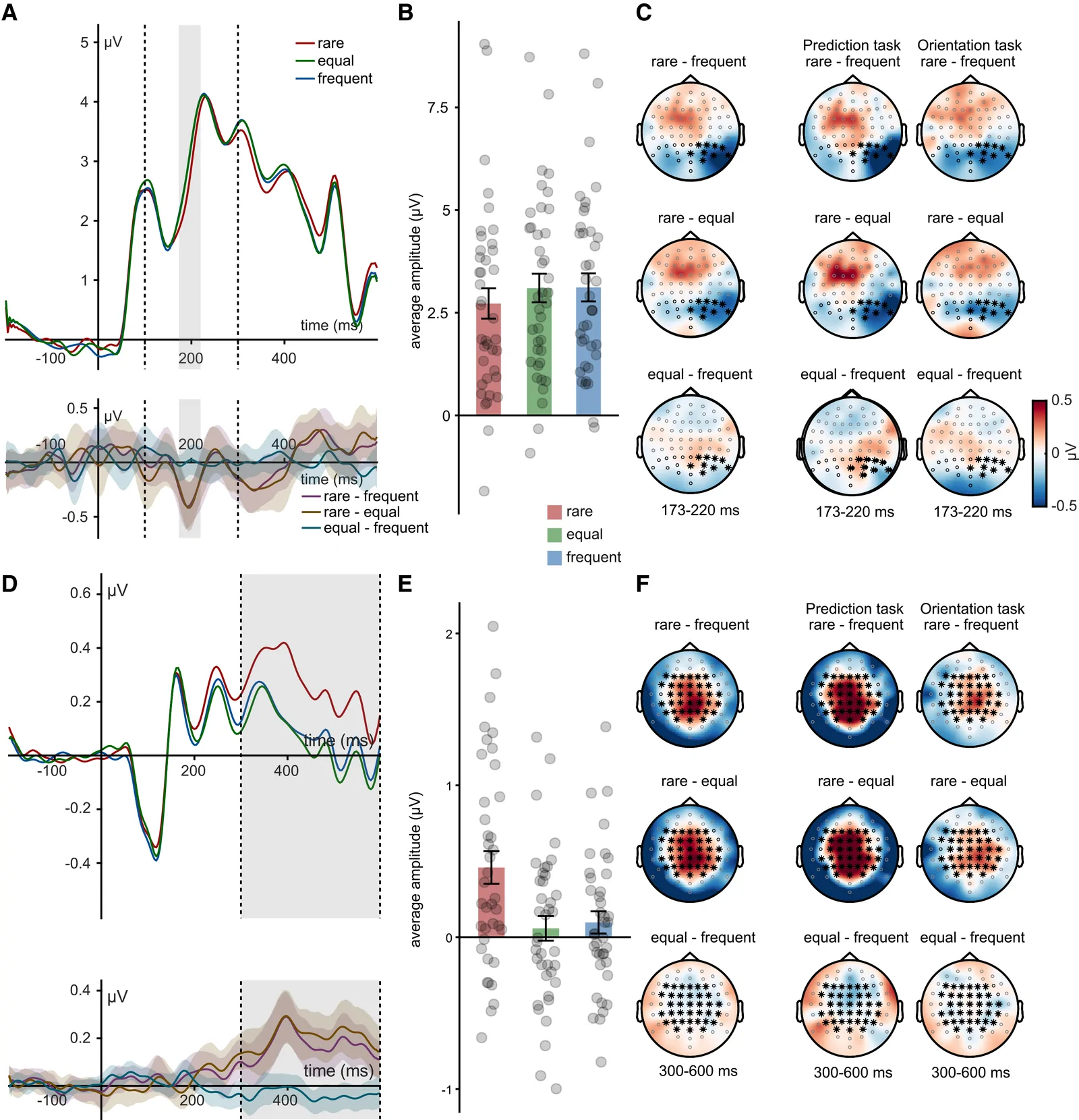
Main effect of stimulus in early and late time window (A) Waveforms and difference waveforms in significant electrodes in the early time window. (B) Cluster averages including single subject data in the early time window. (C) Topographies at significant time points in the early time window. (D) Waveforms and difference waveforms in significant electrodes in the late time window. (E) Cluster averages including single subject data in the late time window. (F) Topographies at significant time points in the late time window. Shaded area marks time points included in the significant cluster; dotted lines indicate the time window included in the cluster-based analysis. The shaded area around difference waveforms depicts the 95%-bootstrap confidence interval. Error bars correspond to standard error of the mean. Asterisks mark electrodes included in the significant cluster, black circles mark electrodes included in the cluster-based analysis, gray circles mark electrodes not included in the analysis.

**Figure 4:**
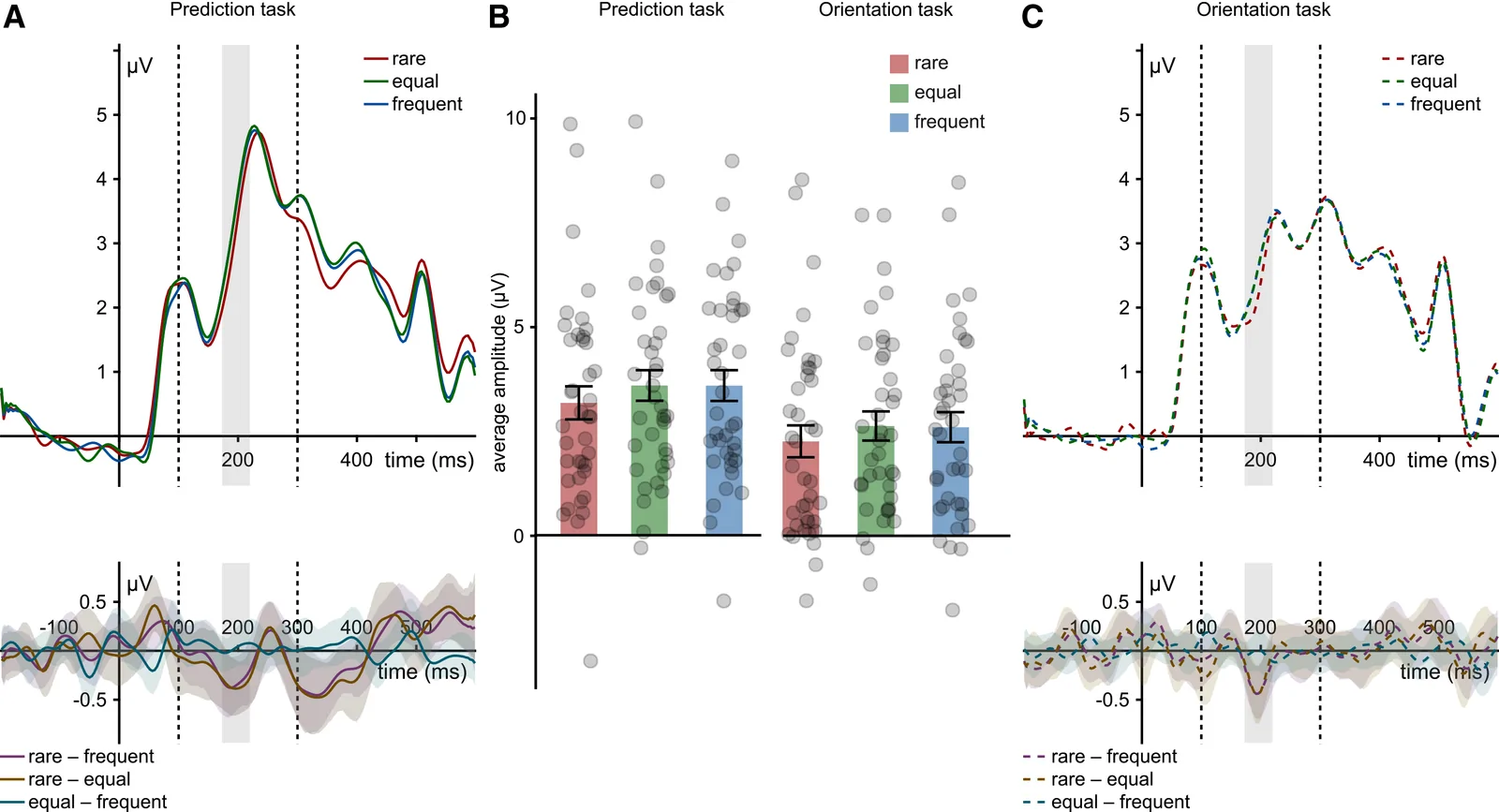
Main effect of Stimulus in the early time window plotted separately for the prediction and orientation task (A) Waveforms and difference waveforms in significant electrodes in the prediction task (solid lines). (B) Cluster averages including single subject data for both tasks. (C) Waveforms and difference waveforms in significant electrodes in the orientation task (dotted lines). Shaded area marks time points included in the significant cluster; dotted lines indicate the time window included in the cluster-based analysis. The shaded area around difference waveforms depicts the 95%-bootstrap confidence interval. Error bars correspond to standard error of the mean. Please note that the corresponding topographies are displayed in Figure 3.

In order to investigate whether the early stimulus effect could be explained by either a more pronounced negativity to rare stimuli or a difference in time-to-peak we extracted peak latencies of the P2 peak. A 2 × 3 repeated measures ANOVA on the latencies indicated no significant effect of neither probability (*F*[2, 68] = 1.37, $\hat{\varepsilon }$ = 0.74, *p* = 0.26), nor task (*F*[1, 34] = 0.15, *p* = 0.70), nor a significant interaction (*F*[2, 68] = 0.30, $\hat{\varepsilon }$ = 0.62, *p* = 0.63).

### Late positivities are potentiated by task relevance

Cluster-based permutations revealed a significant main effect of probability (sum[*F*] = 10,953.86, *p* < 0.001) extending from 300 to 600 ms with a temporal peak at 458 ms. Post-hoc *t* tests indicated that cluster averages of rare stimuli were significantly more positive compared to both frequent and equal condition (rare vs. frequent: *t*[38] = 6.12, *p*_*holm*_ < 0.001, BF_10_ = 41.42×10^3^; rare vs. equal: *t*[38] = 6.19, *p*_*holm*_ < 0.001, BF_10_ = 50.48×10^3^), while evidence for the absence of an effect between equal and frequent condition remained inconclusive (*t*[38] = −1.23, *p*_*holm*_ = 0.23, BF_01_ = 2.89). Importantly, this pattern could be observed in the orientation (rare vs. frequent: *t*[38] = 3.17, *p*_*holm*_ = 0.006, BF_10_ = 11.77; rare vs. equal: *t*[38] = 3.64, *p*_*holm*_ = 0.002, BF_10_ = 37.36; equal vs. frequent: *t*[38] = −0.50, *p*_*holm*_ = 0.62, BF_01_ = 5.16) as well as the prediction task (rare vs. frequent: *t*[38] = 5.46, *p*_*holm*_ < 0.001, BF_10_ = 5850.13; rare vs. equal: *t*[38] = −3.04, *p*_*holm*_ < 0.001, BF_10_ = 9,598.26; equal vs. frequent: *t*[38] = −1.14, *p*_*holm*_ = 0.26, BF_01_ = 3.26). See Figure 3B for a visualization of the results. Furthermore, a significant main effect of task was found (sum[*F*] = 61,175.85, *p* < 0.001) extending from 300 to 600 ms with a temporal peak at 470 ms. Post-hoc *t* tests indicated that cluster averages were more positive in the orientation task than the prediction task (*t*[38] = −11.33, *p*_*holm*_ < 0.001, BF_10_ = 78.77×10^9^). In addition, a significant interaction was found (sum[*F*] = 3,004.56, *p* < 0.001) extending from 300 to 587 ms with a temporal peak at 544 ms. Post-hoc *t* tests indicated that in this cluster the rare, frequent and rare, equal difference was larger in the prediction task than in the orientation task (prediction task – orientation task_rare – frequent_: *t*[38] = 5.14, *p*_*holm*_ < 0.001, BF_10_ = 2,388.61; prediction task – orientation task_rare – equal_: *t*[38] = 5.65, *p*_*holm*_ < 0.001, BF_10_ = 494.76), while there was evidence for the absence of a difference for equal, frequent between tasks (prediction task – orientation task_rare – equal_: *t*[38] = −0.54, *p*_*holm*_ = 0.60, BF_01_ = 5.06). See Figure 5 for a visualization of the results.

**Figure 5:**
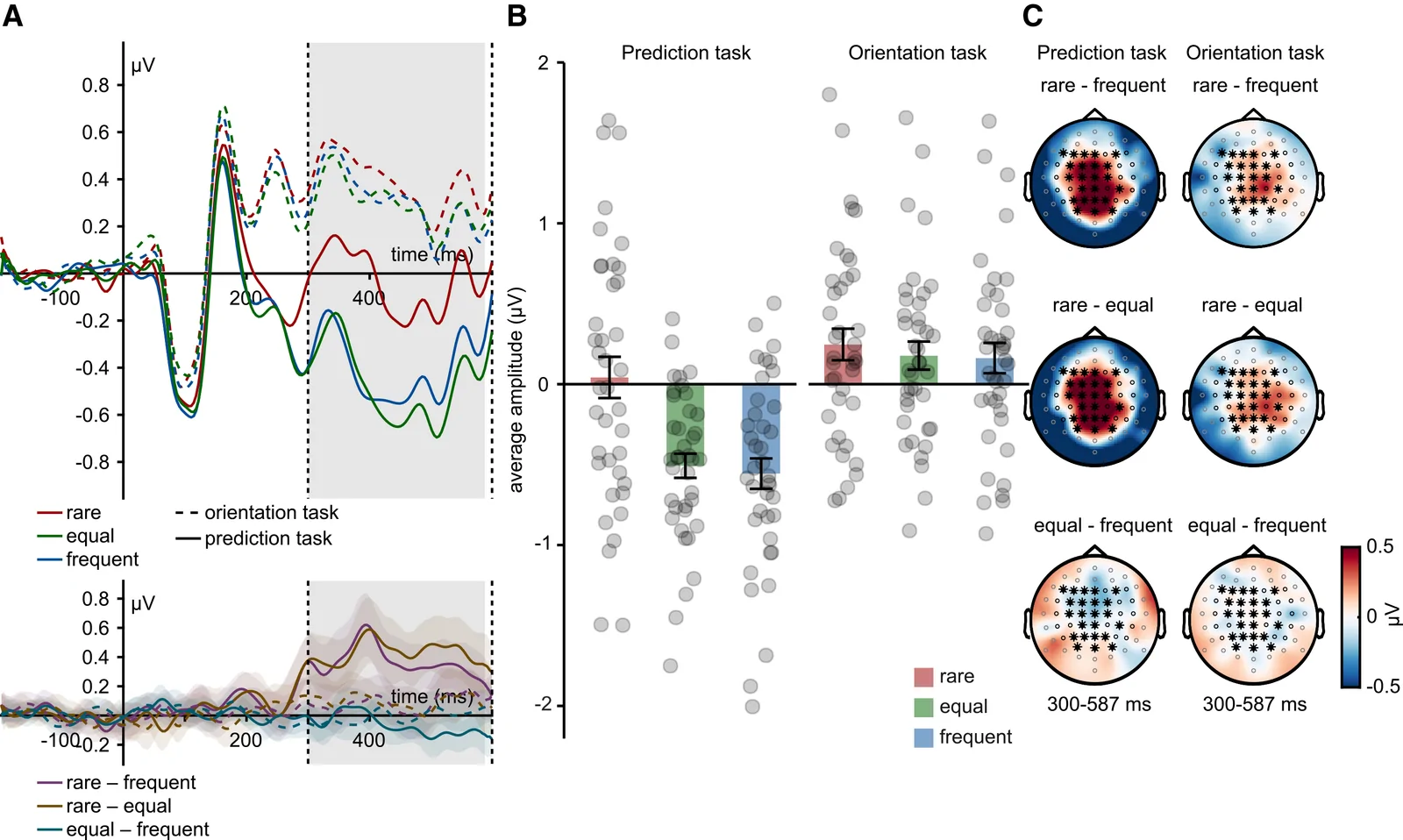
Significant interaction effect between stimulus and task in the late time window (A) Waveforms and difference waveforms in significant electrodes. (B) Cluster averages including single subject data. (C) Topographies at significant time points. Shaded area marks time points included in the significant cluster; dotted lines indicate the time window included in the cluster-based analysis. The shaded area around difference waveforms depicts the 95%-bootstrap confidence interval. Error bars correspond to standard error of the mean. Asterisks mark electrodes included in the significant cluster, black circles mark electrodes included in the cluster-based analysis, gray circles mark electrodes not included in the analysis.

As our analysis especially focused on early negativities and later positivities, we run an additional exploratory cluster-based permutation, including all timepoints after stimulus onset. In the analysis of the posterior electrodes, we recovered a marginal significant cluster for the factor stimulus (sum[F] = 522.50, *p* = 0.095; extending from 173 to 220 ms with a temporal peak at 196 ms) and a significant cluster corresponding in part to the significant late central effect and its opposite pole (sum[F] = 5,503.97, *p* < 0.001; extending from 278 to 600 ms with a temporal peak at 567 ms). Task effects in posterior clusters extended beyond the previously tested time window, with two significant clusters (sum[F] = 4,625.70, *p* = 0.01; extending from 1 to 165 ms with a temporal peak at 134 ms and sum[F] = 42,867.71, *p* < 0.001; extending from 157 to 600 ms with a temporal peak at 243 ms). A significant interaction effect was recovered that corresponded to part of the interaction cluster we reported in the analysis of central electrodes in the late time window (sum[F] = 1,277.73, *p* = 0.005; extending from 376 to 587 ms with a temporal peak at 544 ms). See Figure 6A for a visualization of the effects. In the analysis of the central electrodes, we found a significant cluster for the factor stimulus (sum[F] = 12,022.85, *p* < 0.001; extending from 224 to 600 ms with a temporal peak at 458 ms). For the factor task, we found one significant cluster (sum[F] = 95,211.83, *p* < 0.001; extending from 1 to 600 ms with a temporal peak at 470 ms). Furthermore, for the interaction, we found one significant cluster (sum[F] = 3,121.70, *p* = 0.001; extending from 278 to 587 ms with a temporal peak at 544 ms). See Figure 6B for a visualization of the effects. Furthermore, in order to investigate potential effects of eye movements, we run repeated-measures ANOVA with the factors task and probability on averaged activity of VEOG and HEOG channels at the significant time points of our stimulus effects. Neither main effects nor interactions reached significance (all *p*s > 0.19).

**Figure 6:**
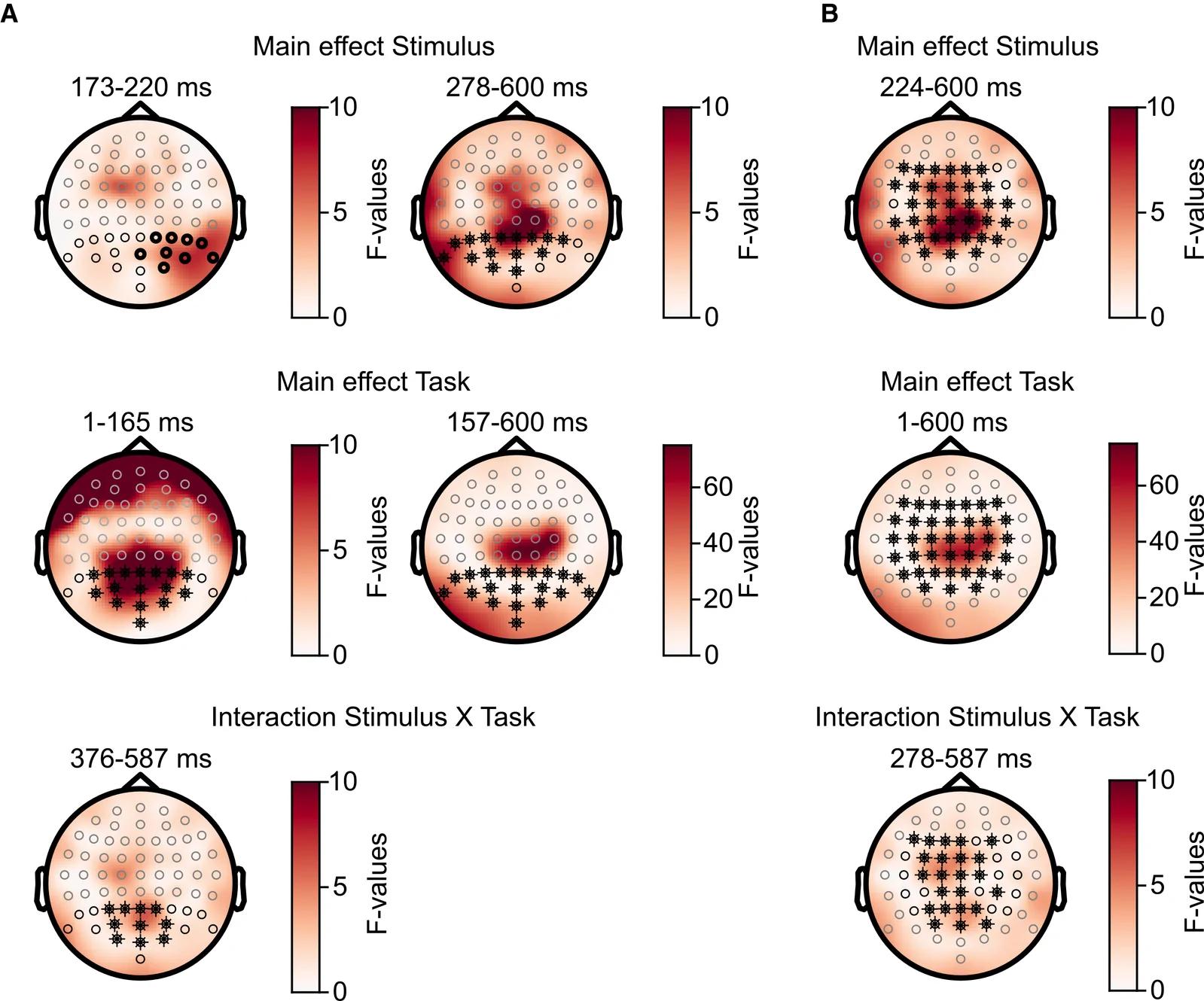
Exploratory analysis of time window from 1 to 600 ms (A) Results in posterior electrodes. (B) Results in central electrodes. Topographies display average *F* values of clusters with *p* < 0.1 for the factors stimulus, task and the interaction of stimulus and task. Asterisks mark electrodes included in significant clusters, bold black circles mark electrodes included in marginal significant clusters, black circles mark electrodes included the cluster-based analysis, gray circles mark electrodes not included in the analysis.

## Discussion

In the present study, we investigated visual probabilistic cueing effects on ERPs during either attentional allocation to the contingencies of cue-stimulus pairs or absent attentional allocation to the pairing. On a behavioral level, participants’ performance indicated that they easily complied with both tasks. Attentional allocation to the predictive relationship affected reaction times to rare and frequent stimuli, whereas attention to the unrelated line orientation did not. Importantly, we found that rare stimuli elicited an increased early negativity and late positivity, and that all probabilistic cueing effects were driven by an enhancement of responses to unexpected stimuli rather than by expectation suppression. Furthermore, we found that the early enhancement was independent of task, whereas later effects were modulated by task-relevance.

Thus, while the presence of probabilistic cueing effects is in accordance with fMRI studies,^7^^,^^8^^,^^9^^,^^10^^,^^11^^,^^12^^,^^27^ and two EEG-MEG studies,^13^^,^^14^ it contrasts with several studies.^15^^,^^16^^,^^17^^,^^18^ Remarkably, we found evidence for probabilistic cueing effects in an early time window across right hemispheric visual areas, even in the condition in which cue-stimulus contingency was not task-relevant. While the lateralization to the right hemisphere was unexpected, it is in line with the results of a recent review on the visual MMN that reported a right lateralization in 78% of studies that found lateralized effects.^22^ There is evidence for a functional specialization in regard to the type of stimulus that is being processed, i.e., visuospatial attention vs. language,^28^ which might in turn also affect probabilistic cueing effects. At a first glance, the effect size of this early effect appears rather small and might represent a delayed response rather than a difference in amplitude due to its occurrence in between two peaks. However, the size of the effects is about 0.5 μV for the early as well as the late effect (see e.g., difference waveforms in Figure 3) and the amplitude difference of approximately ∼ −0.5 μV is in line with other work on probabilistic cueing.^14^ Furthermore, additional analysis revealed no significant difference in time-to-peak between conditions indicating the presence of a genuine difference in amplitudes.

Possible reasons for the clarity of our findings could lie in our simple design, which used a clear mapping of only two stimuli, each predicted by three different cues, allowing clarity about contingencies during the experiment. In other studies, more complex probabilistic relationships were used,^15^^,^^16^^,^^18^ or additional types of expectancy, such as motor expectancy, were manipulated.^17^ This aligns with work highlighting the importance of the uniqueness of the prediction as compared to simple conditional probabilities.^29^ Furthermore, we used a line-orientation task that is orthogonal to cued shapes, avoiding complex or interfering attention tasks. For example, in den Ouden et al.,^15^^,^^16^ the task introduced a third stimulus in the sequence that could either repeat or alternate, which may have confused the perception of rare and frequent stimulus presentations. These tasks similarly led to at least some attention to the cued stimulus, while the probabilistic relationship to the cue was irrelevant.^15^^,^^16^^,^^18^ In another study, Hu et al.^17^ showed no effects, even if the probabilistic relationship was relevant to the task. In contrast, our data and those of two other studies^13^^,^^14^ show enhanced responses to unexpected stimuli in task-irrelevant conditions.

The presence of probabilistic cueing effects despite attention to an unrelated feature could indicate that the processing of these probabilistic relationships occurs at least somewhat automatically like the MMN.^30^ Given the inconclusive evidence about whether or not probabilistic cueing effects can be observed in the visual modality, we decided for a stimulus setup that was as simple as possible. In doing so our design also resembled the design of MMN studies more closely. In this field that stimulus set and predictive relationships are usually more restricted.^23^ Thus, we can link our findings much more easily to the MMN literature. Thus, it seems that at least similar procedures with restricted stimulus sets might result in similar findings in MMN and probabilistic cuing studies. However, it remains unclear whether the scope of the current results is indeed influenced by the restricted stimulus set and simple set up and our study can be seen as a starting point that lays out a foundation for further systematic research on probabilistic cueing effects on visual ERPs.

Concerning the underlying mechanisms of early effects, comparisons to the neutral condition revealed that they were driven by surprise enhancement, i.e., PE, while evidence against an expectation suppression effect was uncovered. This is in line with previous studies^12^^,^^13^^,^^20^ and with evidence for a genuine MMN driven by surprise enhancement, i.e., a PE, during early time windows.^23^ It has been hypothesized that attention might especially enhance responses to unexpected stimuli, thereby shifting the balance among the underlying mechanisms of cue-based learning.^6^ However, it has to be noted that this singular PE-like signal in the absence of expectation suppression, that is, the reduced activation to predicted stimuli, does not support some influential predictive coding accounts, which assume that both phenomena should be observed.^1^^,^^2^^,^^3^^,^^4^^,^^5^ Furthermore, while there is some evidence of task relevance impacting the degree of predictive processing in oddball paradigms,^25^ our pattern of results during probabilistic cueing is not in line with this hypothesis, showing that the underlying mechanism at this early time point is not influenced by an attentional manipulation. Thus, the nature of this perceptual surprise signal has to be established in future studies.

In our study, we not only uncovered evidence for early probabilistic cueing effects but also effects in a later time window, which is in line with previous EEG studies identifying expectation effects in a later time window.^31^^,^^32^^,^^33^ Furthermore, here, we observed a potentiated late positivity to rare stimuli, with significantly stronger effect in the task-relevant condition than in the irrelevant condition. This is in line with findings showing an enhancement in P3 components with increasing relevance of stimuli.^24^^,^^34^ In oddball designs, the P3 has been associated with PE processing^35^^,^^36^^,^^37^ and model updating.^34^ Thus, in contrast to the early perceptual PE, from the perspective of predictive coding accounts, the P3 can be seen as a PE response at a later stage in the processing hierarchy,^38^^,^^39^ that enables updating the model of the world and subsequent model adjustment.^35^^,^^36^^,^^37^ Furthermore, in the predictive processing framework, the enhanced surprise signal present in the prediction task might represent the higher precision of the prediction brought about by attention to the predictive relationships, and thus a higher PE if the prediction is violated.^1^^,^^2^^,^^40^ However, there are alternative explanations for an enhanced response that should be considered.

For example, the reaction time data during the prediction task might indicate that the rare condition was more difficult than the frequent condition as its response time was significantly longer. It has been shown that the P3 is influenced by task difficulty and decision making,^41^^,^^42^^,^^43^ which might have impacted the current findings. However, if the P3 response only indexes the task difficulty approximated through response time, it should also be enhanced for the equal probability condition. However, the opposite was the case, amplitudes in the equal probability condition resembled the frequent condition despite longer reaction times.

All in all, the results of our study might at least partially be integrated into the hierarchical predictive processing framework,^1^^,^^2^^,^^3^^,^^4^ showing an early process indexing a PE response that is less dependent on task-relevance than later stages, which may indicate PE on a higher hierarchical level and updating processes. However, given that we uncover evidence for the absence of expectation suppression, these findings might pose a challenge to the theory.^6^ Because the amount of expectation suppression and surprise enhancement has not been explicitly formalized, it remains unclear whether the hierarchical predictive processing framework can account for the absence of expectation suppression.

In summary, we observed significant visual probabilistic cueing effects and found that early PE-related responses are present irrespective of task, while later effects are modulated by task relevance. Irrespective of timing and task, probabilistic cueing effects were driven by an enhancement of responses to unexpected stimuli and not by expectation suppression. Thus, our results are in line with the proposals of hierarchical predictive processing that posit that unexpected events are accompanied by a PE whose processing level is linked to the attentional state of the participant.

### Limitations of the study

The study comes with certain limitations. While the clarity of our findings shows that probabilistic cueing effects occur in both relevance conditions, future research is needed to investigate whether this holds true when participants are distracted by a demanding other task or when stimuli are presented unaware. Furthermore, we used a small number of cues, fixed contingencies and a specific stimulus configuration. To generalize findings, it may be useful to take a stepwise approach to manipulate the size of the stimulus set and complexity of probabilistic relationships directly. Furthermore, while we did not detect systematic eye movement artifacts in our data, it might nevertheless be worthwhile to include eye tracking in a future study to investigate this issue more closely and to exclude eye movements as an alternative explanation for our findings. Concerning behavioral findings, we only observed effects of the probability manipulation on performance in the prediction task, while this might indicate that only in this task behavior was influenced, potential effects could also have been masked due to ceiling effects. Future studies should calibrate the tasks to allow for effects in all conditions. Lastly, we did not preregister our analysis plan, e.g., time windows for statistical tests, which would have strengthened our results. Exploratory analysis in the whole time window lined up, at least as tendencies, with the effects reported in the manuscript while potentially highlighting a slightly earlier onset of the late stimulus and interaction effects. They also showing the limits of the cluster-based ANOVA approach where effects of opposite polarity might be included in the same cluster (see e.g., late stimulus effect in posterior electrodes that includes the positive as well as negative pole of the effect). Furthermore, the time window and electrode selection of our primary analysis were motivated by previous results in MMN research.^24^^,^^25^ This enabled us to enhance the power of the cluster-based permutation, especially in the context of previous null-findings. Nevertheless, future studies should use preregistrations to further investigate the reliability of probabilistic cueing effects.

## Resource availability

### Lead contact

Requests for further information and requests should be directed to and will be fulfilled by the lead contact, Insa Schlossmacher (insa.schlossmacher@uni-muenster.de).

### Materials availability

This study did not generate new unique reagents.

### Data and code availability

• Raw data reported in this study will be shared by the lead contact upon request. Preprocessed data has been deposited on the Open Science Framework and is publicly available at OSF: https://doi.org/10.17605/OSF.IO/PUQE5 as of the date of publication.
• All original code has been deposited on the Open Science Framework and is publicly available at OSF: https://doi.org/10.17605/OSF.IO/PUQE5 as of the date of publication.
• Any additional information required to reanalyze the data reported in this study is available from the lead contact upon request.

## Acknowledgments

We would like to thank all participants for their time and effort.

## Author contributions

I.S.: conceptualization, investigation, formal analysis, methodology, software, visualization, and writing – original draft; A.S.: formal analysis, investigation, and writing – original draft; S.S.: conceptualization, investigation, formal analysis, methodology, visualization, and writing – original draft; R.M.: investigation, software, and writing – review and editing; T.S.: conceptualization, methodology, supervision, and writing – review and editing.

## Declaration of interests

The authors declare no competing interests.

## STAR★Methods

### Key resources table

**Table:** 

REAGENT or RESOURCE	SOURCE	IDENTIFIER
**Deposited data**		

current study data	Study cohort	OSF: https://doi.org/10.17605/OSF.IO/PUQE5

**Software and algorithms**		

MATLAB 2021a	MathWorks	RRID:SCR_001622
R Version 4.4.2	R Foundation	RRID:SCR_001905
Psychophysics Toolbox 3	http://psychtoolbox.org/	RRID:SCR_002881
FieldTrip	https://www.fieldtriptoolbox.org/	RRID:SCR_004849
Factorial Mass Univariate Toolbox	https://github.com/ericcfields/FMUT/wiki/	RRID: SCR_028612
current study code	study	OSF: https://doi.org/10.17605/OSF.IO/PUQE5

### Experimental model and study participant details

The sample consisted of 40 participants (29 female) aged from 20 to 29 years (*M* = 23.50, *SD* = 2.08). All had normal or corrected-to-normal vision, and one was left-handed. Participants volunteered and were compensated with 12 € per hour. Before starting, participants were given written instructions on the experimental task, given the opportunity to ask further questions and provided written informed consent. The study was approved by the ethics committee of the German Psychological Association (Deutsche Gesellschaft für Psychologie, DGPs, No.: SchlossmacherInsa2021-02-28VADM).

### Method details

#### Apparatus

The experiment was run using MATLAB 2021a and the Psychophysics Toolbox 3.0.19.^44^^,^^45^^,^^46^ A Dell P2419H monitor (Dell, Texas, USA) at 60 Hz with a resolution of 1920 × 1080 pixels was employed for stimulus display. The viewing distance amounted to 60 cm. To respond, participants pressed the space bar and arrow keys of a standard keyboard.

#### Stimulus material and experimental procedure

Stimuli consisted of circles with a radius of 1.6 degrees of visual angle (°) and rectangles, spanning 6.9 ° × 3.45 ° presented on a dark gray background (0.2). The line width for all stimuli amounted to 0.8°. Circle outlines could either be green, orange or blue ([90,212,95], [208,114,13], or [101,168,201] in RGB), while rectangle outlines were always black. The area inside of the circle was background colored, while the insides of the rectangles did contain a sinewave pattern with a spatial frequency of 1° and a contrast of 1 that was either tilted 45° to the left or right (Figure 1A). Different colored circles were used as cues for either vertical or a horizontal rectangle with a contingency of either 80:20 horizontal to vertical, 80:20 vertical to horizontal, or 50:50 vertical to horizontal. For example, for one participant, the green circle would represent an 80% probability, the orange circle would represent a 20% probability, and the blue circle a 50% probability of a horizontal rectangle (Figure 1B). Allocation of colors to rectangles were counterbalanced across participants. Gabor patch orientation was orthogonal, such that left or right-tilted Gabor patches were presented in equal parts in rare, frequent, and equal probability trials.

One trial always started with the presentation of a fixation cross for 1000 ms, followed by the presentation of a circle for 400 ms, followed by an interstimulus interval of on average 500 ms (±100 ms), followed by the presentation of a rectangle for 400 ms. In 20% of the trials, another interstimulus interval of on average 500 ms (±100 ms), the current task question was presented to the participants (Figure 1A).

Before the experiment, participants were explicitly informed about the contingencies between the circle’s colors and the rectangle form. For both experimental conditions, the stimulus presentation was physically identical, while the task differed. Furthermore, they completed 16 practice trials before starting each task. In the prediction task, participants had to pay attention to the contingencies between colored circles and rectangles. In 20% of trials, they had to answer the question ‘Which rectangle did you expect after the orange/blue/green circle?’ by choosing from a vertical and horizontal rectangle presented on the screen. In the orientation task, participants had to pay attention to the tilted Gabor patches inside the rectangles. In 20% of trials, they had to answer the question ‘In which direction were the lines tilted?’ by choosing from right and left-tilted square Gabor patches on the screen. The order in which participants completed the tasks was counterbalanced across participants, i.e., half of the participants started with the prediction task while the other half started with the orientation task. In total, 480 trials were presented in randomized order with equal amounts of trials for each cue circle. After 160 and 320 trials, there was a self-paced break. The runtime of one condition amounted to approximately 25 min (excluding breaks).

#### EEG recording and preprocessing

A 64-channel BioSemi active electrode system (BioSemi B.V., Amsterdam, Netherlands) was employed to collect electrophysiological data. Electrodes were placed using the 10/20 system conforming to BioSemi electrode caps. Instead of ground and reference, the BioSemi EEG system uses a CMS/DRL feedback loop with two additional electrodes (for more information see: https://www.biosemi.com/faq/cms&drl.htm). Electrical potentials were recorded with a sampling rate of 512 Hz, and impedances were held below 20 kΩ. A built-in analog anti-aliasing low-pass filter of 104 Hz was applied prior to digitization.

Preprocessing of the EEG data was performed using the FieldTrip toolbox^47^ in MATLAB. Offline filtering of the continuous data employed Butterworth filters with half-power cut-offs if not specified otherwise. Data were band-stop filtered at 49–51 Hz (roll-off: −24 dB/octave) and at harmonic frequencies (up to 199–201 Hz) in order to minimize line noise. A 0.1 Hz high-pass filter (roll-off: −12 dB/octave) removed slow drifts. Then, the EEG signal was segmented into epochs of 200 ms before stimulus onset until 600 ms after stimulus onset. Using an independent component analysis (ICA), horizontal and vertical eye movements were removed from the data. Trials containing muscle artifacts and electrode jumps were manually excluded based on visual inspection and bad channels were interpolated. Data were re-referenced from the CMS/DRL to a common average reference. All trials were baseline-adjusted using the average of a prestimulus interval from −200 to 0 ms.

ERP responses to rectangles of each participant were averaged separately for rare, frequent, and equal probability. This resulted in six waveforms (rare/frequent/equal for the prediction and orientation task) per participant. Lastly, grand mean waveforms of the averaged data were computed.

### Quantification and statistical analysis

Statistical analysis employed a factorial, mass-univariate approach in combination with cluster-based permutation (CBP) tests.^48^ We included the factors task and probability in a 2 × 3 design. In order to enhance the power of the cluster-based analysis, we chose specific time intervals and electrode locations based on previous research. For the analysis of the early negativities, we chose a time interval from 100 ms to 300 ms in posterior electrodes,^24^^,^^25^ see Figure 3A. For the late positivity, we selected centro-parietal electrodes and an interval from 300 ms to 600 ms,^34^ see Figure 3B. In an exploratory analysis, we additionally included all time points so that we did not miss effects outside of our time-windows of interest. Clusters were formed by two or more neighboring sensors (in time and space) whenever the *F*-values exceeded the cluster threshold (α = 0.05). The cluster mass, sum(*F*), was calculated by adding all *F*-values within a cluster. The number of permutations was set to 5000, and the significance value for testing the null hypothesis amounted to α = 0.05. Prior to the analysis, ERPs were down-sampled to 256 Hz and low-pass filtered at 25 Hz (roll-off: −24 dB/octave) to further enhance statistical power.^49^ After applying the CBP approach, cluster averages from significant clusters were computed and used to investigate significant main and interaction effects. Then, we used two-sided *t*-tests with the Bonferroni-Holm correction method to compare cluster averages. While the difference in trial numbers does not affect our cluster-based permutation approach as it does not assume equal variance between conditions, we still investigated how it impacted the variance by running Mauchly’s test for sphericity on the extracted cluster averages.

In order to test whether the early effect represented a delayed response to rare stimuli we run a time-to-peak analysis by extracting the peak latency of the P2 peak for each participant in each condition between 150 and 300 ms in electrodes indicated in the significant cluster. The peak finding algorithm as implemented in MATLAB (*findpeaks*) was able to obtain a peak latency in all conditions for 35 of our participants. After extraction, we run an 2 × 3 repeated measures ANOVA on the latencies. Furthermore, we computed VEOG and HEOG and averaged depending on task and probability condition. We run repeated measures ANOVAs on averaged activity in significant time windows of our stimulus effects.

For the prediction task, behavioral performance was analyzed by extracting the percentage of matches between the shown rectangle and the corresponding button press and the median reaction time for each probability condition (rare, frequent, equal). Again, for the orientation task, we extracted the hit rate and median reaction time for each probability condition. Percentages and median reaction times were analyzed using repeated-measures ANOVAs and two-sided *t*-tests. Whenever sphericity was violated, the Greenhouse-Geisser correction was applied, and corrected *p*-values as well as $\hat{\varepsilon }$-values are reported below. As some of our conclusions rely on null effects, we additionally report Bayes Factors (BF), with BF_01_ denoting the evidence for the null hypothesis and BF_10_ for the alternative hypothesis. We use the conventions from Jeffreys^50^ to interpret the results of our Bayesian analyses. All cluster-based permutations were done using the Factorial Mass Univariate Toolbox (FMUT).^51^ Other statistical tests relied on the statistics program R.^52^
